# Effect of mammalian mesopredator exclusion on vertebrate scavenging communities

**DOI:** 10.1038/s41598-020-59560-9

**Published:** 2020-02-14

**Authors:** Kelsey L. Turner, L. Mike Conner, James C. Beasley

**Affiliations:** 1 University of Georgia Savannah River Ecology Laboratory, Warnell School of Forestry and Natural Resources, P.O. Box Drawer E, Aiken, SC 29802 USA; 2 The Jones Center at Ichauway, 3988 Jones Center Drive, Newton, GA 39870 USA; 3 Present Address: USDA-APHIS-WS, 400 Northeast Dr Suite L, Columbia, SC 29203 USA

**Keywords:** Behavioural ecology, Food webs

## Abstract

Carrion is a valuable resource used by facultative scavengers across the globe. Due to conflicts with humans, many vertebrate scavengers have experienced population declines due to direct persecution or indirect effects of human activities. However, little is known about the implications of altered scavenger community composition on the fate and efficiency of carrion removal within ecosystems. In particular, mammalian mesopredators are efficient scavengers that are often subjected to control, thus, it is important to understand how the reduction of this scavenger guild influences the fate of carrion resources and efficiency of carrion removal within ecosystems. We evaluated the influence of the absence of mammalian mesopredators on vertebrate scavenging dynamics by comparing the efficiency of carrion removal and species composition at carrion between sites where we experimentally manipulated mesopredator abundance and paired control sites. Overall scavenging rates were high, even within our mesopredator exclusion sites (79% of carcasses). Despite the exclusion of an entire guild of dominant scavengers, we saw little effect on scavenging dynamics due to the extensive acquisition of carrion by avian scavengers. However, we observed a slight reduction in vertebrate scavenging efficiency in sites where mesopredators were excluded. Our results suggest vertebrate communities are highly efficient at carrion removal, as we saw a functional response by avian scavengers to increased carrion availability. These data provide insights into the impact of mesopredator control on food web dynamics, and build upon the growing body of knowledge investigating the role of vertebrate scavengers on ecosystem services provided through carrion removal.

## Introduction

Scavenging ecology is a complex yet understudied area of ecology^1–3^. Carrion provides a valuable nutritive resource to a diversity of organisms found in all biological kingdoms^1,3–5^. To vertebrate scavengers, carrion is an important resource, and unless predated and consumed whole, vertebrates inevitably supply this resource through their own natural deaths^1,3^. Although terrestrial vertebrate scavenging was traditionally thought to be dominated by obligate scavengers (i.e., vultures), there is increased recognition that most vertebrates are facultative scavengers, including species not usually associated with carrion^5,6^. Facultative scavengers are well documented taking carrion globally^7,8^ and often dominate acquisition of small to mid-sized carrion^9–13^, yet little is known about the explicit role of many facultative scavengers in food web dynamics.

Previous studies have shown disruptions to the abundance or composition of vertebrate scavengers can have dramatic effects on local scavenging communities and ecosystem processes. For example, the reintroduction of gray wolves (*Canis lupus*) in Yellowstone National Park has helped sustain other facultative scavenger populations through winter when food is limited by supplying a steady source of ungulate carrion^14–17^. The regular sustenance of wolf-killed carcasses has resulted in increased fitness of facultative scavenger populations, which has increased competition at carcasses and led to increased hunting pressure by wolves on abundant ungulate populations in Yellowstone^14^.

Conversely, the loss of a top scavenger can have detrimental consequences to carcass persistence and nutrient cycling, as the efficiency of carrion removal by vertebrates is explicitly linked to the composition and distribution of scavengers^18–20^. Thus, anthropogenic disturbances that alter scavenger community dynamics can have profound impacts on the fate of carrion^10,19,21,22^. For instance, the removal of dominant scavengers, such as vultures, likely played a role in the population growth of feral dog and rat populations in parts of Asia due to reduced competition for carrion resources^23^, resulting in substantial impacts to human health due to increased incidence of rabies^24^. Furthermore, carcasses have also been documented as reservoirs or potential transmission sites for a number of other diseases globally such as chronic wasting disease^25^, black plague (*Yersinia pestis*^26^), tuberculosis^27^, anthrax, and canine distemper virus^28^. While all scavengers provide an important role in removing carrion from the environment, vultures, in particular, are especially important not only because of their efficiency at locating carrion, but also because direct and indirect disease spread to other scavengers is limited at carcass sites where vultures are present^18,23,29^.

The loss of facultative scavengers within ecosystems also may negatively influence the overall efficiency of the scavenger community. For example, Hujibers *et al*.^30^ observed a decrease in the number of carcasses successfully located and scavenged by vertebrates in areas where predacious birds, top scavengers in that system, were absent. Similarly, Olson *et al*.^10^ observed a 42% increase in the rate of unscavenged mouse carcasses following a reduction in the raccoon (*Procyon lotor*) population, a dominant scavenger in that ecosystem. Mammalian mesopredators, such as raccoons, are highly efficient facultative scavengers that can rapidly assimilate the majority of carrion in landscapes supporting high densities of these species^31^. Consequently, abundant mesopredators have been demonstrated to outcompete other mammalian and avian facultative scavengers for carrion resources in some ecosystems^10^. Thus, intentional or unintentional shifts in the abundance of mesopredators or the composition of this important scavenging guild could create an imbalance in the efficiency of dead matter removal.

Given the impact observed following the reduction of a single facultative scavenger from an ecosystem^10,20,30^, removal of an entire guild of mammalian predators may yield a dramatic change in scavenger efficiency in the remaining community, which, to date, has not been evaluated. Studying shifts in the composition of predator guilds has important implications, as predator control and persecution is a widespread practice globally^32–34^ and the effects of predator control on nutrient recycling via scavenging dynamics are poorly understood. Predator management techniques may dramatically alter the scavenging community and have unintended consequences on ecosystem services provided by scavenging predators. To elucidate the effect of predator control on carrion fate, we experimentally evaluated the effect of mammalian mesopredator exclusion on the efficiency of carcass removal as well as the composition of other scavenger guilds present within the ecosystem. We hypothesized carcasses available to all guilds would be discovered faster than those restricted from mesopredators, more carcasses would be lost to decomposition in the absence of mesopredators, and that more carcasses excluded from mesopredators would be taken by avian scavengers than those available to all guilds.

## Materials and Methods

### Study area

**Figure 1:**
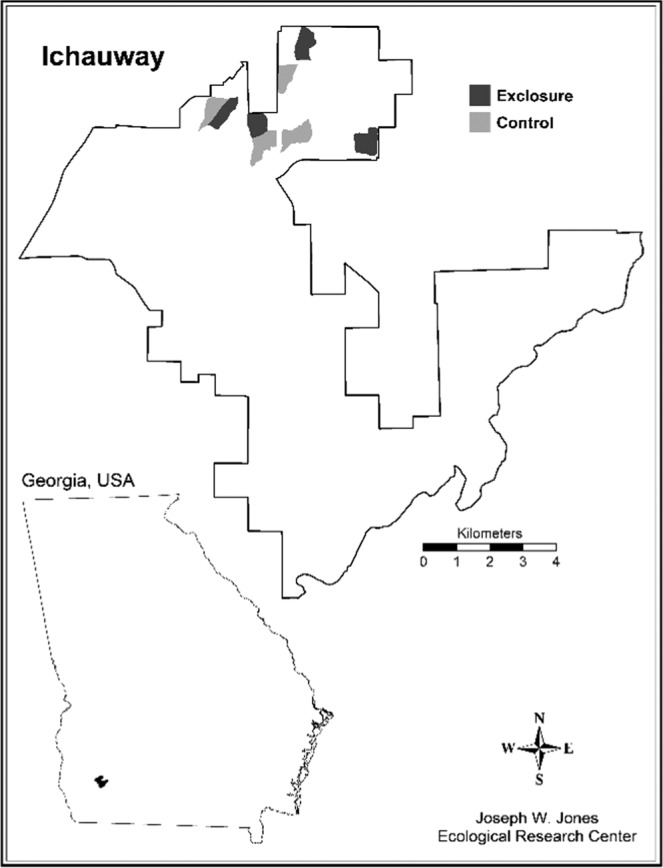
Study area map indicating the distribution of habitats across Ichauway, a 11,736 ha plantation located in southern Georgia, in the southeastern U.S. Ichauway is composed of a variety of habitats including hardwood, agricultural fields, natural long leaf (*Pinus palustris*) forest, other pine forest, mixed forest, and shrub/scrub. Fenced exclosures and associated unfenced, control sites are located in northern Ichauway. Map was created using ArcGIS 10.3.1 (http://www.esri.com).

This research was conducted at the Jones Center at Ichauway (Ichauway), located in the southwestern coastal plain of Georgia, U.S. The site was created in the early twentieth century for quail hunting but is now managed for both game production and conservation. Ichauway is 11,736 ha and fire-maintained, with over half the site burned under a bi-annual low intensity fire regimen^35^. Ichauway receives an average of 141 cm of rainfall each year and average yearly maximum and minimum temperatures are 25.2 °C and 11.9 °C, respectively^36^. Although surrounded by agricultural land, Ichauway is dominated by a longleaf pine (*Pinus palustris*) wiregrass (*Aristida* spp.) ecosystem but also includes riparian hardwoods and swamps, flatwoods, pine stands (i.e., *Pinus taeda* and *Pinus elliottii*), as well as agricultural fields^35^ (Fig. 1). Ichawaynochaway Creek runs through the site while the Flint River outlines Ichauway’s eastern boundary.

Ichauway is home to numerous mammalian mesopredator species, including raccoons, gray fox (*Urocyon cinereoargenteus*), red fox (*Vulpes vulpes*), bobcat (*Lynx rufus*), Virginia opossums (*Didelphis virginiana*), striped skunks (*Mephitis mephitis*), and coyotes (*Canis latrans*)^37,38^. Based on track count surveys conducted along transects during Summer 2014, these species, excepting skunk, were observed on Ichauway during our study (Fig. S1, unpublished data). Four exclosures, each approximately 40 ha and surrounded by a four-foot fence with 10 × 20 mesh and three strands of high-voltage electric wires (Fig. S2), were constructed on Ichauway between 2002 and 2003 to experimentally exclude mesopredators^37–39^. Although highly effective in excluding most mesopredators, individuals are occasionally able to gain access to exclusion sites^37,38^. Routine track count surveys within the controls and exclosures during our study revealed that while mesopredators were reduced within exclosures, raccoons and opossums were occasionally observed (Fig. S3, unpublished data). Therefore, fences were actively maintained and any mesopredators discovered inside, based on camera images or animal sign, were captured and removed. Four unfenced control sites, each approximately 40 ha, were also established in between 2002 and 2003.

### Field methods

We conducted scavenging trials monthly from July 2014 through June 2015 in each of the exclosures and the unfenced control areas. Trials consisted of a single rabbit (*Oryctolagus cuniculus*) carcass placed in each of the eight study sites every month at a randomly chosen location. For all scavenging trials we staked down medium-sized (0.9–1.8 kg) rabbits ordered frozen from an online source (RodentPro.com, LLC, Inglefield, IN) to mimic the average size of an adult eastern cottontail rabbit (*Sylvilagus floridanus*; 0.8–1.5 kg^40^), a common prey species within our study site. Only brown, “wild looking” rabbits were used to eliminate any bias due to coat coloration variation. We thawed rabbits indoors at room temperature prior to deployment in the field. This research was carried out in accordance with all relevant guidelines and regulations established through the University of Georgia Institutional Animal Care and Use Committee (IACUC). All field methods and experimental protocols were approved under the University of Georgia IACUC protocol A2014 04-023-Y3-A1.

We monitored carcasses with Reconyx PC900 Hyperfire^TM^ infrared, no-glow remote sensing cameras (RECONYX, Inc., Holmen, WI). Each camera was deployed for 30 days, which included a 15-day trial length and a 15-day lag period to buffer trials. The lag period was added to reduce the possibility of wildlife becoming habituated to carcasses being supplied at sites. We assumed 15 days to be a sufficient lag period based on the removal of similar-sized rabbit carcasses in a previous study in the region^12^. We programmed cameras to take a burst of 3 pictures when triggered by motion with a 30 second quiet period between bursts. Additionally, because we wanted to document decomposition in the absence of vertebrate scavengers, we also programmed cameras to take a time lapse photo every 30 minutes. Carcass remains (i.e., hair and bone) were removed at the end of the 30 days to remove visual attractants and reduce bias to subsequent trials. Subsequent trials were placed at random locations at a minimum distance >100 m away from the previous trial location.

No cameras were deployed in February 2015 due to routine prescribed fire in the study sites. Cameras were placed again in March, 2 weeks post-fire. We assumed 2 weeks between the low-intensity fire event and camera placement was enough time for wildlife to resume normal behavior^41^. Because air temperature has known effects on carcass decomposition^1,12,42,43^, we categorized camera data between two seasons defined as a “warm” and “cool” based on temperature. We delineated the warm season (July-October 2014 and April-June 2015) based on the hottest months with average temperatures above 15.5 °C, whereas the cool season (November 2014-March 2015) was delineated based on months with average temperatures below 15.5 °C^36^.

### Camera analysis

Images were downloaded from cameras at the end of each trial and analyzed to determine: (1) the identity, date, and time of the first vertebrate scavenger and all subsequent visitors, (2) carcass interaction type (i.e., scavenged or not), (3) the number of individuals of a given species utilizing carcasses at a given visit, (4) date and time each species left the carcass site, (5) the identity of the final scavenger and the date and time at which it left, and (6) date and time of full depletion of carcass by microbes and invertebrates if the final vertebrate scavenger did not fully scavenge that carcass. We defined a visit as at least one representative of a species being present with no absence surpassing 5 minutes. Because scavengers would exit a camera’s field of view repeatedly during a visit and our inability to distinguish individuals, we considered extended periods of absence in determining visitation duration to account for the possibility of a vertebrate being flushed away from a carcass, even briefly. If the same species appeared on camera after that elapsed 5-minute absence period, the interaction was counted as a new visit. These data were used to assess carcass fate, frequency of occurrence for all vertebrate scavengers, scavenger species richness, detection time, and carcass removal time.

### Statistical methods

We analyzed differences in the fate of carrion, elapsed time to detection, vertebrate scavenger community composition, and carcass removal across treatment types and seasons. We used R Version 3.4.3^44^ to perform all analyses. Carcasses prematurely taken from the camera’s field of view (n = 2) were removed from all analyses.

#### Carcass fate

We assessed carcass fate by quantifying the number of unscavenged and scavenged carcasses for each season and treatment. We defined a “scavenged” carcass as any carcass that was partially or fully scavenged by vertebrates. Eighty-six trials were included in this analysis; Carcass fate was categorized as either being scavenged or not, and frequencies were calculated for both treatments and seasons. We conducted a log-linear analysis of the frequency of carcass acquisition as a function of treatment type, season, and carcass fate (i.e., yes or no) using a Poisson generalized linear model with a log link^44^. Two models were constructed to investigate (1) the 3-way interaction of the fixed effects treatment, season, and carcass fate and (2) the 2-way interaction of significant fixed effects (i.e., season and carcass fate). Models evaluating significant individual main effects were not needed.

#### Detection time

We defined detection time as the time elapsed between when the carcass was placed and when the first vertebrate scavenger discovered the carcass. We analyzed detection time as a function of treatment type and season using generalized linear mixed-effects models (GLMM) using the LME4 package^45,46^. We constructed 4 models, including a null model, using an exponential distribution and inverse link (Table S1). We used the Shapiro-Wilk Normality Test and Q-Q Plots to assess normality and homoscedasticity. The data were log transformed to conform to normality assumptions of GLMM. Carcasses not found by vertebrate scavengers prior to being fully scavenged by invertebrates were censored from this analysis (n = 9), as no flesh remained to be scavenged. We used AIC values to rank and determine supported models (≤2 ∆AIC).

#### Scavenger species composition

We assessed scavenger species composition two ways: (1) calculating percent occurrence for each vertebrate scavenger species documented feeding at carcass sites (i.e., number of carcasses at which a species occurred divided by total number of carcasses), and (2) evaluating difference in vertebrate scavenger richness between exclosure and control sites. Unscavenged carcasses were excluded from this analysis (n = 11).

Scavenger species richness was calculated as the number of scavenger species observed consuming carrion at each carcass. We constructed four generalized linear mixed effects models (GLMM) (Table S2) using a Poisson distribution and a log link (LME4^45^) to analyze scavenger species richness as a function of treatment type and season (fixed effects). Carcass site was included as a random effect. We used AIC values to rank and determine supported models (≤2 ∆AIC). We used a likelihood ratio test (ANOVA) to compare competitive models.

#### Carcass removal

We defined carcass removal as the time elapsed between carcass placement and either full removal by a vertebrate scavenger or complete decomposition. We constructed 4 GLMM models, including a null model, (Table S3; LME4^45,46^) to analyze carcass removal as a function of treatment and season using an exponential distribution and inverse link. We excluded failed trials (n = 2). We assigned decomposition time as carcass removal time to partially scavenged and unscavenged carcasses. We used the Shapiro-Wilk Normality Test and Q-Q plots to assess normality; data were log-transformed to meet normality assumptions of GLMM. We included treatment and season as fixed effects and study site as a random effect. We ranked models based on AIC values and considered all models within 2 ∆AIC as competitive.

## Results

We conducted 88 camera trials (44 trials of both treatment types) resulting in >185,800 images between July 2014 and June 2015.

### Scavenger efficiency

We were able to successfully document carcass fate in 86 of 88 total trials. Overall, 87.2% of successful trials were scavenged by vertebrates; 90.5% (n = 42) within control sites and 84.1% (n = 44) within exclosures. Carcass acquisition also varied by season, as 100% (n = 32) of carcasses were scavenged during the cool season as opposed to 79.6% (n = 54) in the warm season. Seasonal trends were also reflected between treatments during each season—84.6% (n = 26) and 75.0% (n = 28) in warm season controls and exclosures, respectfully. Mesopredators scavenged 25.0% (n = 11) of the carcasses placed within exclosures.

The 2-way interaction between season and carcass fate was the only significant interaction in our log-linear analysis (p < 0.0009). Scavenging rates were higher during the cool season, with 100% (n = 32) and 79.6% (n = 54) of carcasses scavenged by vertebrates in the cool and warm seasons, respectively. Further evaluation of treatment revealed it was not significant (p = 0.83) in determining whether a carcass is scavenged.

### Detection time

We observed 89.5% of carcasses were detected by vertebrate scavenger species, and 97.4% of detected carcasses (n = 77) were then scavenged. Carcasses were discovered by representatives of various vertebrate scavenger species, the most common species to first detect carcasses being turkey vultures (44.2%, *Cathartes aura*), Virginia opossums (27.3%, *Didelphis virginiana*), and red tailed hawks (27.3%, *Buteo jamaicensis*). Overall, avian scavengers were first to detect 68.8% of successfully located carcasses while mesopredators detected 29.9% of carcasses first—averaging 65.7 ± 8.1 hrs and 42.4 ± 8.2 hrs to detection, respectively.

Trends were similar for trials conducted in control sites (N = 40). Avian scavengers (65.0%, 61.1 ± 9.5 hrs) discovered a greater number of carcasses with longer detection times in comparison to mesopredators (35.0%, 33.7 ± 8.4 hrs). Interestingly, carcass detection between mesopredators and the avian guilds were most similar during the cool season (43.7% and 56.2%, respectively) in controls. In contrast, mesopredators, on average, found carcasses much faster than their avian competitors (38.6 ± 14.0 hrs and 94.4 ± 20.4 hrs, respectively). Concurrently, avian scavengers were able to detect over double the amount of carcasses in the warm season in comparison to mesopredators (70.8% and 29.2%, respectively) albeit in similar detection times (43.5 ± 7.0 hrs and 28.9 ± 8.9 hrs, respectively).

Average detection times varied across seasons and treatments, although the seasonal trend was more apparent (Fig. S4). The overall average detection time of carcasses by vertebrate scavengers was 58.6 ± 6.2 hours (n = 77). During the warm and cool seasons, average detection times were 38.4 ± 4.2 hrs (n = 45) and 86.9 ± 12.1 hrs (n = 32), respectively. Detection times ranged from 1.0 to 110.5 hrs in the warm season and 1.6 to 261.3 hrs during the cool season. Additionally, warm season control and exclosure carcasses were detected by vertebrates in an average 39.3 ± 5.7 (n = 24) and 37.4 ± 6.3 (n = 21) hrs, respectfully. During the cool season, control and exclosure carcasses were found in an average of 70.0 ± 14.5 (n = 16) and 103.8 ± 18.8 (n = 16) hrs, respectfully—a 33.8 hr difference. Overall, average detection times for controls and exclosures were 51.6 ± 7.0 (n = 40) and 66.1 ± 10.3 hrs (n = 37), respectively—a 14.6 hr difference.

Our GLMM analysis of detection yielded three competitive models (Table S2). The null model and the model including only the fixed-effect season were equally ranked top models (∆AIC = 0, *w* = 0.80), suggesting that while temperature trends were apparent, the model was not strong enough in determining detection time across carcasses. Additionally, the third-ranked model containing only the variable treatment was also competitive (∆AIC = 2, *w* = 0.15). However, this model only represented a model weight of 0.15 and the variable treatment also fell out in the lowest ranked model (∆AIC > 3) suggesting treatment was an uninformative variable.

### Scavenger species composition

Eight vertebrate species were documented scavenging during our study (n = 65) (Table 1). Scavenger species documented included representatives of the avian (turkey vulture; black vulture, *Coragyps atratus*; crow, *Corvus* spp.; red tailed hawk, *Buteo jamaicensis*; red shouldered hawk, *Buteo lineatus*; great horned owl, *Bubo virginianus*) and mesopredator (Virginia opossum; raccoon, *Procyon lotor*) scavenging guilds. A single gopher tortoise was observed attempting to scavenge an unopened carcass. Overall, the species most frequently documented scavenging included turkey vultures (54.7%, n = 47), Virginia opossums (33.7%, n = 29), and red tailed hawks (20.9%, n = 18). These three species dominated carrion consumption in the control sites (Table 1), whereas in the exclosures the most frequently occurring scavengers were turkey vultures and red tailed hawks. Black vultures were also documented scavenging in both the exclosures and control sites, but at a much lower frequency than turkey vultures. Surprisingly, raccoons were only documented at four carcasses. Occurrence of turkey vultures was consistent between treatments, occurring more frequently during the cool season (62.5%) than the warm season (50.0%) in both treatments. Red tailed hawks, however, were observed more variably across treatments and seasons, occurring most often in the exclosures during the cool season (43.6%).

**Table 1:** Percent (%) frequency of occurrence of scavenger species observed consuming carcass bait presented across treatment types (mesopredator exclusion and control) and seasons based on carcass trials completed in July 2014 – June 2015 at Ichauway in southwest Georgia, USA.

Scavenger Species	Control		Exclosure		Overall % Occurrence (n = 86)
	Warm (n = 26)	Cool (n = 16)	Warm (n = 28)	Cool (n = 16)	
**Avian**					
Turkey vulture	50.0	62.5	50.0	62.5	54.7
*Cathartes aura*					
Red tailed hawk	26.9	12.5	7.1	43.6	20.9
*Buteo jamaicensis*					
Black vulture	7.7	6.3	10.7	6.25	8.1
*Coragyps atratus*					
Red shouldered hawk	0.0	6.3	3.6	0.0	2.3
*Buteo lineatus*					
Crow	3.8	0.0	0.0	0.0	1.2
*Corvus* spp.					
Great horned owl	3.8	0.0	0.0	0.0	1.2
*Bubo virginianus*					
**Mammal**					
Virginia opossum	42.3	56.3	21.4	18.6	33.7
*Didelphis virginiana*					
Raccoon	0.0	6.3	3.6	6.3	4.7
*Procyon lotor*					

Overall, average scavenger species richness at a carcass was 1.45 ± 0.09 (n = 75; range 1–4); median species richness was 1. Our GLMM analysis yielded three competitive models (ΔAIC < 2) (Table S2). The null model, which contained no fixed effects, was the top model, although models including treatment (ΔAIC = 0.6) and season (ΔAIC = 0.6) were also supported.

Average species richness was similar between the warm 1.44 ± 0.11 (n = 43) and cool 1.47 ± 0.13 (n = 32) seasons, which suggests the third ranked model containing season was not biologically relevant. Average species richness was similar between treatments, although richness was slightly greater at control sites 1.55 ± 0.12 (n = 38) than at treatment sites 1.35 ± 0.12 (n = 43). The majority of carcasses in exclosures (62.8%) were consumed by a single scavenger, and only 18.6% of carcasses were scavenged by two species. Carcasses in control sites were most often scavenged by a single (55.3%) or 2 scavengers (36.8%). Overall, only 5 carcasses (6.7%, n = 75) were scavenged by more than two species.

### Carcass removal

Average carcass removal time was 97.8 ± 7.9 hrs (n = 86) across our combined study sites. Our GLMM analysis yielded one competitive model containing the variable season (Table S3). Treatment repetitively fell out in the worst models (∆AIC > 3) suggesting the presence of mesopredators was uninformative in determining removal times of carcasses (Fig. S5).

Average removal time in the warm season was 60.4 ± 4.2 hrs (n = 54), compared to 160.8 hrs ± 14.3 (n = 32) in the cool season. Removal times were similar between treatments, as the average among control carcasses was 98.4 ± 12.1 hrs (n = 42) compared to 97.2 ± 10.3 hrs (n = 44) in the exclosures. Average removal time differed by 7.32 hrs between the control and exclosure treatments during the cool season (164.4 ± 23.2 hrs, n = 16 and 157.1 ± 17.6 hrs, n = 16, respectively) but differed by 5.23 hrs between controls (57.7 ± 4.2 hrs, n = 26) and exclosures (62.9 ± 7.1 hrs, n = 28) during the warm season.

## Discussion

We observed high rates of carcass acquisition by vertebrates, even within sites excluding mammalian mesopredators (79%), supporting previous studies demonstrating vertebrates are highly efficient at assimilating small carrion resources^12,31,43,47^. Despite the almost complete exclusion of an entire guild that extensively utilizes medium-sized carrion^12,48^, our study suggests vertebrate scavenging communities, as a whole, are resilient to shifts in species composition, especially in landscapes with intact avian scavenging guilds. However, previous studies excluding obligate scavengers (i.e., vultures) have reported increased persistence time of carrion or an incomplete functional response by facultative scavengers^19,23^. Such discrepancies in the functional response of vertebrate scavengers to shifts in community composition suggest scavenging dynamics may be most sensitive to disruptions in populations of obligate scavengers. Nonetheless, mean time to detection of carcasses in our study was 14.6 hours longer and carcass acquisition rates by vertebrates was reduced by 15.9% within exclosures compared to our control sites, suggesting that while the remaining scavenging community efficiently removed carrion within exclosures, they were not as efficient as in areas containing intact scavenging communities. Although this reduction in efficiency was not statistically significant in our models, the resulting impact on decomposers and nutrient cycling may be biologically relevant^43^.

Facultative scavengers intensively compete both amongst themselves as well as with obligate scavengers for carcass resources, although most scavenging by facultative scavengers is likely opportunistic^6,23^. Thus, the composition of vertebrate scavengers and efficiency of those species may be dynamic through space and time with shifts in species abundance or foraging behavior, yet overall efficiency of carrion removal may remain similar. Our results support this supposition as rates of carcass detection, removal, and scavenger species richness were relatively similar between treatment and control sites, suggesting other guilds (i.e., obligate and facultative avian scavengers) in our system were able to increase scavenging rates in the absence of mammalian mesopredators. Indeed, we observed a marked increase in scavenging by red-tailed hawks in exclosures during the cool season, suggesting a functional response by this species in the absence of mammalian scavengers.

We also observed temporal differences in scavenging dynamics similar to patterns reported in other studies^3,31,42,43,48^. In particular, carcass acquisition rates by vertebrates differed substantially between seasons, ranging from 79.2% in the warm season to 100% in the cool season. This seasonal shift in acquisition likely reflects increased activity of microbes and invertebrates during the warm season^1,21^, a pattern further supported by an increase in carcass removal times during the cool season. Ultimately, although it took scavengers longer to locate carcasses during the cool season, likely due to decreased olfactory cues^4,42,43,49^, scavengers remained able to successfully acquire 100% of carcasses. Thus, even in the absence or reduction of decomposer activity, vertebrates were highly effective at detecting and consuming carrion experimentally placed on the landscape, even in our mesopredator exclosures.

The overall composition of the vertebrate community observed in this study likely contributed to the similarities in carcass removal between controls and exclosures, as carcasses were located at similar rates and ultimately scavenged by a similar group of scavengers in both treatment and control sites. Specifically, turkey vultures, opossums, and red tailed hawks dominated carcass acquisition, and both avian species scavenged at high frequencies in both exclosures and control sites. We originally hypothesized more carcasses would be scavenged by birds in exclosures due to the absence of mesopredators, but did not anticipate high frequencies of facultative avian scavengers in the controls as well^12^. We suggest the dominance of scavenging in control sites by highly efficient avian scavengers overshadowed any potential impact of mesopredator exclusion on rates of carrion detection and removal. Thus, scavenging dynamics and the competitive balance between decomposer and vertebrate scavenging communities—for access to scavenging-derived nutrients—may be less affected by perturbations to facultative vertebrate scavenger populations in landscapes supporting robust populations of obligate scavengers or efficient facultative avian scavengers. Nonetheless, while there was a strong trend in carcass acquisition by avian scavengers in the absence of mesopredators, the avian scavenging community could not entirely compensate for the lack of mesopredator scavenging, largely driven by high scavenging rates of Virginia opossums at our control sites. This response is similar to a previous study which experimentally reduced raccoon populations to assess the response of the remaining scavenging community^10^. While other species were observed taking more carrion, especially Virginia opossums, the number of unscavenged carcasses increased following the reduction in raccoon numbers, suggesting mesopredator control has implications to scavenging dynamics^10^.

Despite the diversity of scavengers that occur at Ichauway, we observed a limited number of species scavenging (n = 8) compared to other studies using similar carcass sizes (n = 16^48^; n = 13^12^), despite similarities in community diversity^12,47,49–52^. Interestingly, not only did we observe a truncated vertebrate scavenging community, we also unexpectantly observed a limited diversity of mesopredators (i.e., raccoons and opossums) scavenging at our control sites despite a diverse assemblage of mesopredators (i.e. coyotes, gray foxes, bobcats, and raccoons) that Ichauway supports^38,50–53^. Although availability of alternative food sources could have influenced the limited diversity of mammalian mesopredators observed scavenging in our control sites^31,54^, we also speculate these observations reflect efficient foraging by avian scavengers in our study area. Concurrently, the decrease in detection times and increase in detection by avian scavengers during the warm season may also be attributed to the increased dietary requirements or increased home range sizes of breeding avian scavengers to feed nestlings, thus being observed scavenging more often than mammalian competitors^55–58^.

## Supplementary information

Supplementary information
