# Supplementary material for: Effect of mammalian mesopredator exclusion on vertebrate scavenging communities
Source: Sci Rep. 2020 Feb 14;10:2644. doi: 10.1038/s41598-020-59560-9 (PMC7021701; doi:10.1038/s41598-020-59560-9)
Supplement: Supplementary file 1 — Supplementary information [file 41598_2020_59560_MOESM1_ESM.docx]

**Supplementary Information File**

**Title:** Effect of mammalian mesopredator exclusion on vertebrate scavenging communities

**Authors:** Kelsey L. Turner, L. Mike Conner, & James C. Beasley

Figure S1. Figure depicting the average number of track sets for each mammalian mesopredator species observed per transect line during the Summer 2014 track count survey conducted on Ichauway (averaged over 4 days) in southern Georgia, USA. Figure is based on a 95% CI (based on 1.96*SE).


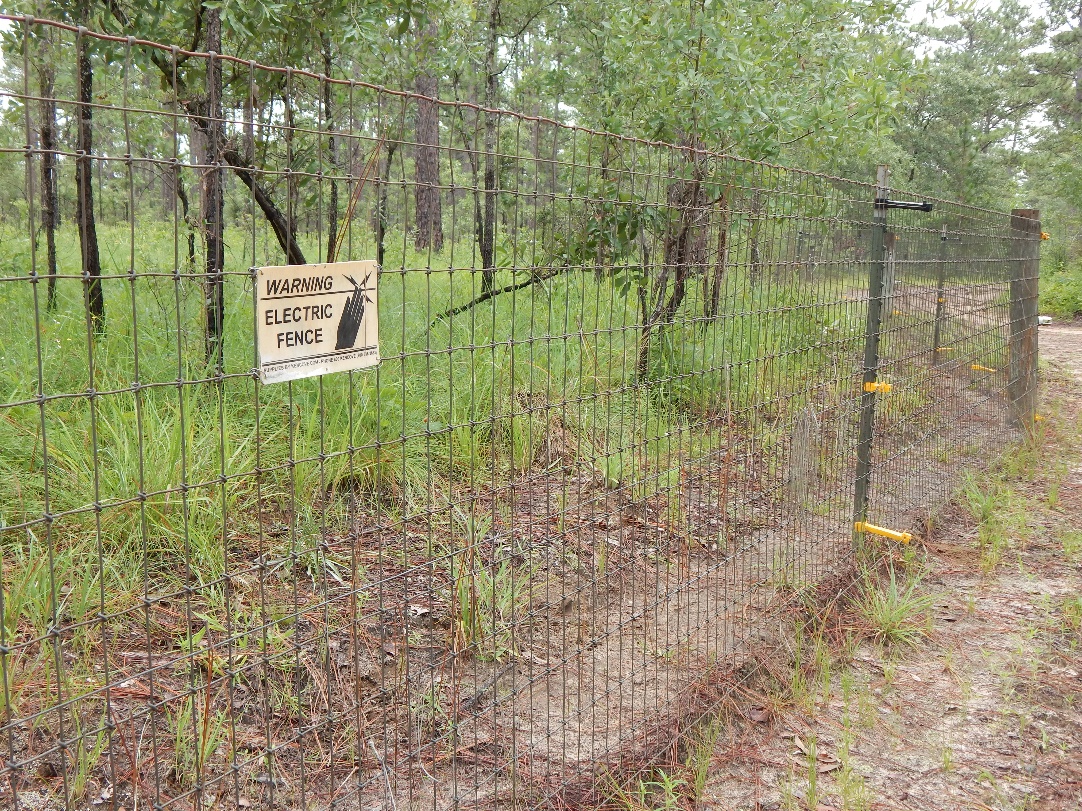


Figure S2. Each of the experimental 40-ha exclosures were surrounded by a four-foot fence with three strands of high-voltage electric wires.


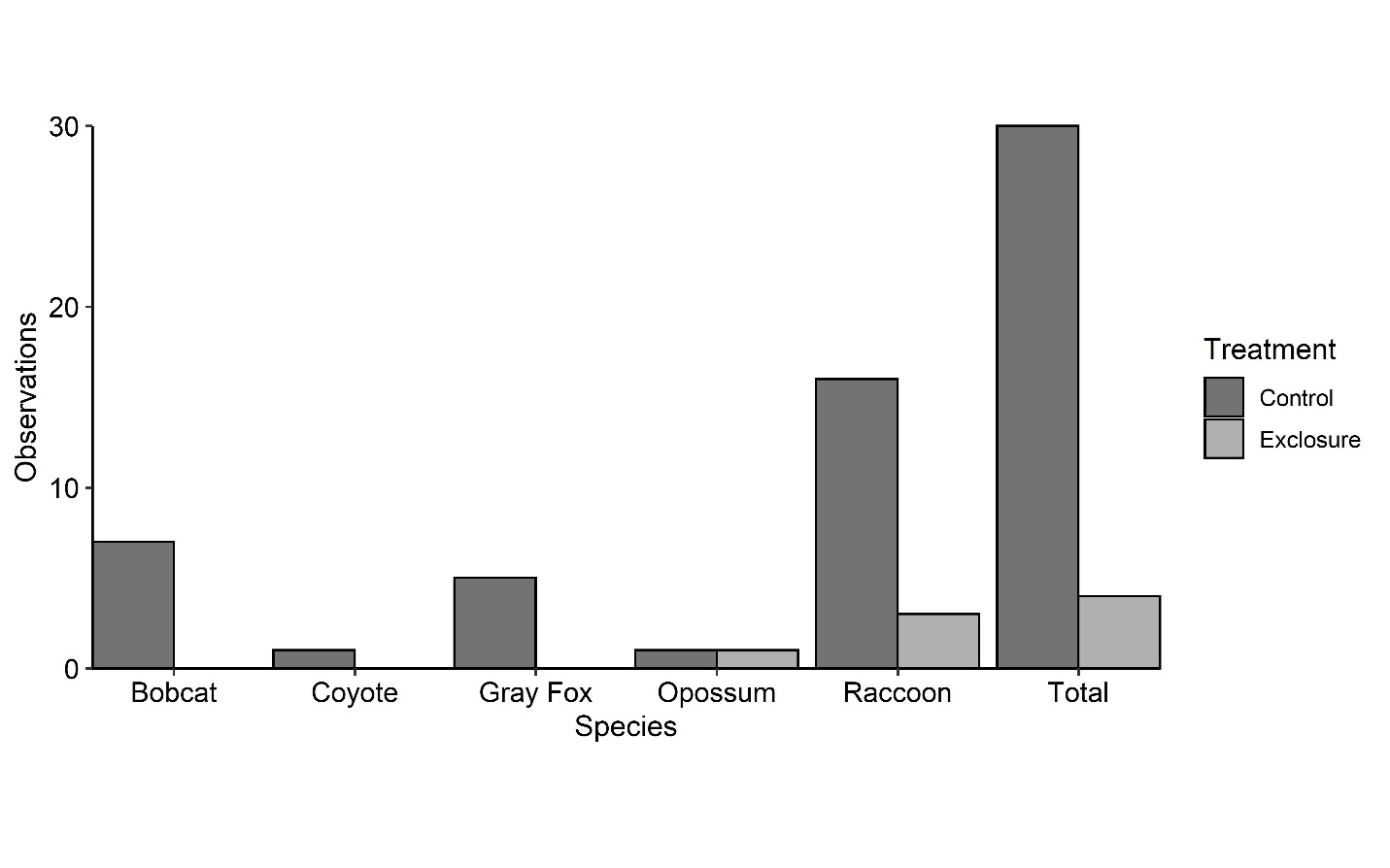
Figure S3. Figure depicting the number of observations of track sets for each mammalian mesopredator species observed across 5 track stations for each control and exclosure within during Summer 2014 – Spring 2015 on Ichauway, Georgia, USA.


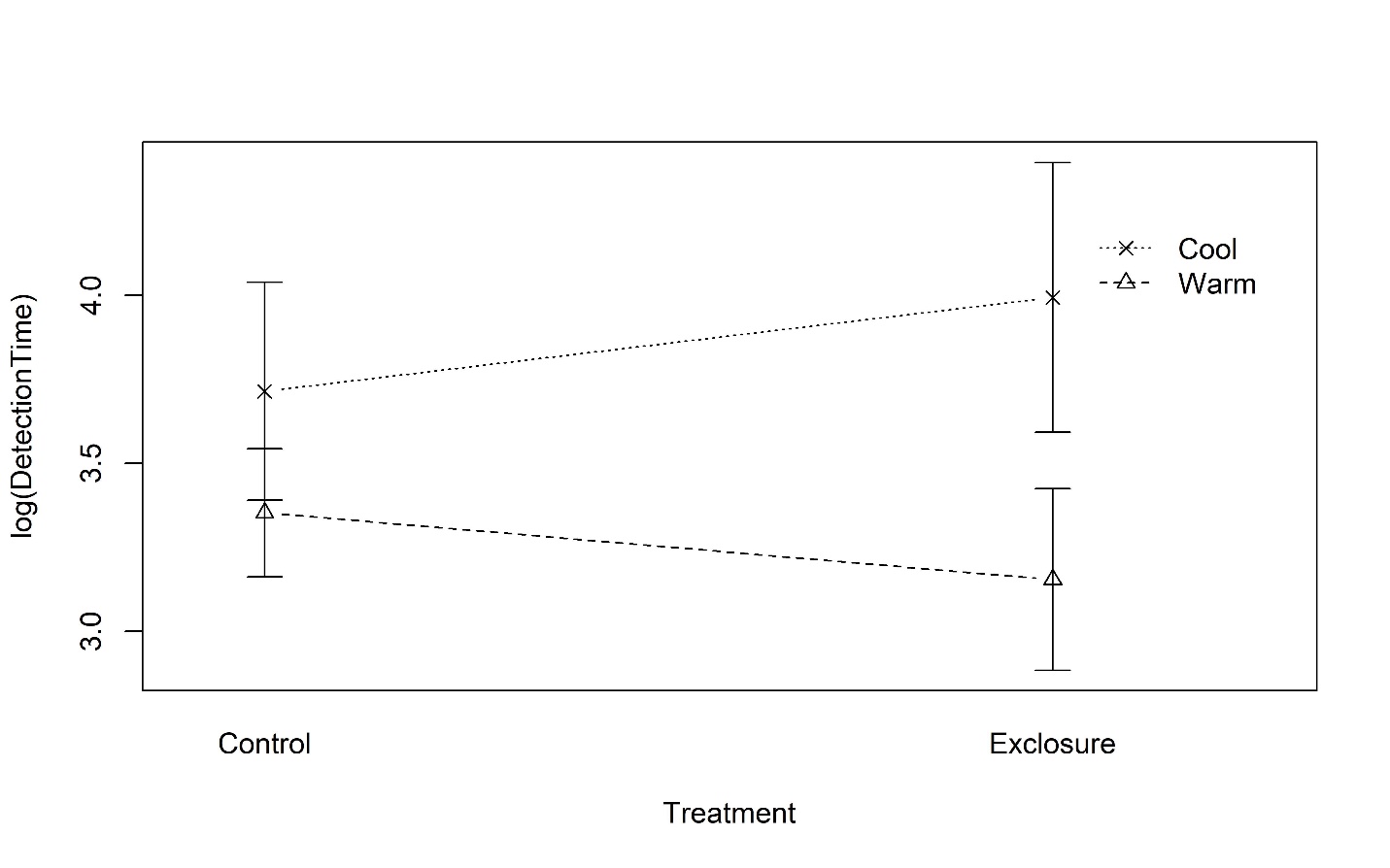


Figure S4. Mean response plot depicting differences in carcass detection time of as a function of season and treatment.


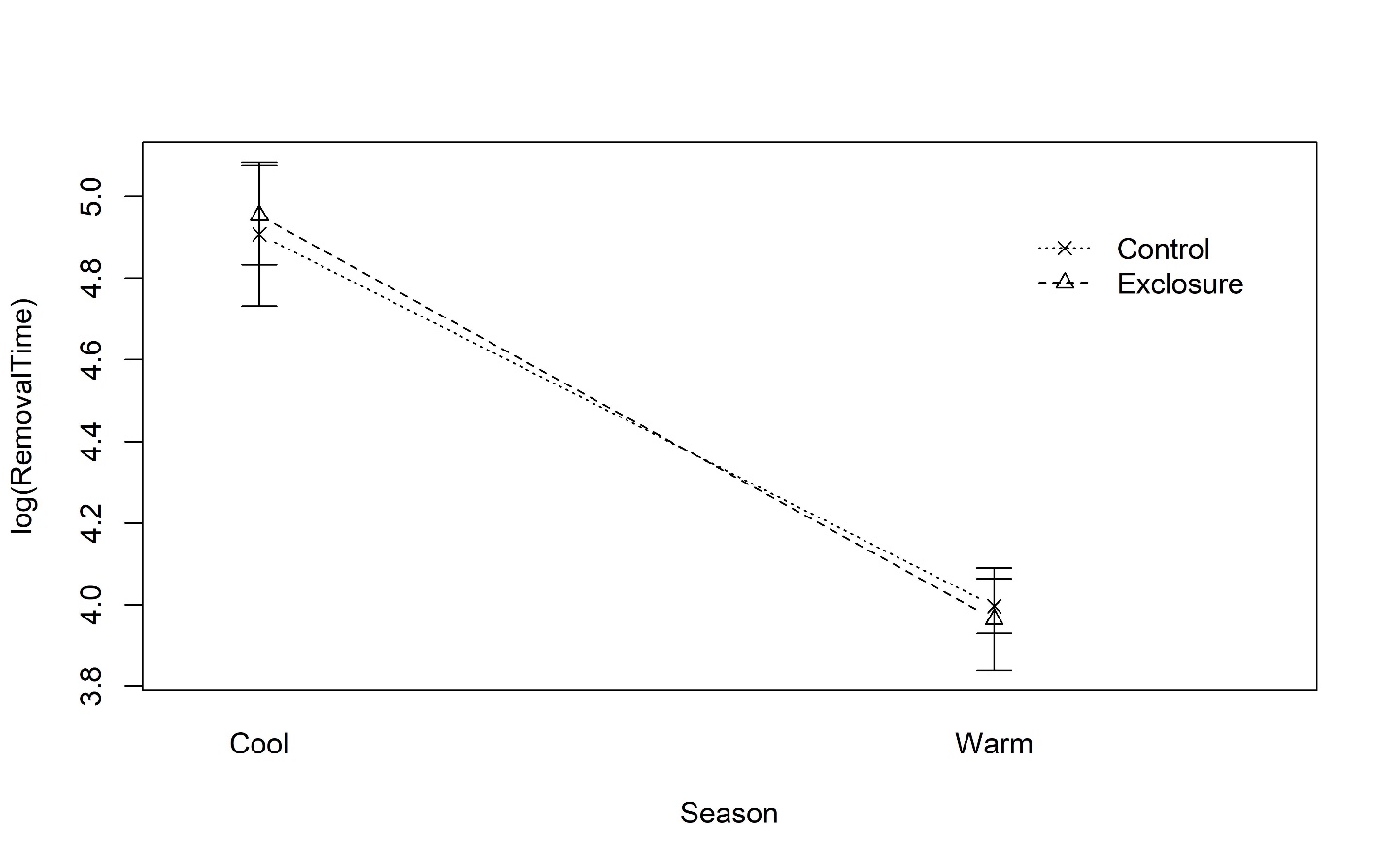


Figure S5. Mean response plot depicting differences in carcass removal time of as a function of season and treatment.

Table S1. Four generalized linear mixed-effects models (GLMM) were constructed with an exponential distribution to analyze differences in carcass detection times as a function of treatment^†^ and season^‡^ based on trials completed from July 2014 – June 2015 in mesopredator exclosures and control sites in each of two seasons in southern Georgia, USA.

| Model | AIC | ∆AIC | *w* | Deviance |
| --- | --- | --- | --- | --- |
| Site(intercept) | 304.4 | 0.0 | 0.40 | 298.4 |
| Season + Site(intercept) | 304.4 | 0.0 | 0.40 | 296.4 |
| Treatment + Site(intercept) | 306.4 | 2.0 | 0.15 | 298.4 |
| Treatment * Season + Site(intercept) | 308.1 | 3.7 | 0.06 | 296.1 |

^†^mesopredator exclosures and controls

^‡^warm and cool

Table S2. Generalized linear mixed effects models (GLMM) constructed to analyze scavenger species richness across treatment^†^ and season^‡^ based on carcass trials completed in July 2014 – June 2015 at Ichauway in southwest Georgia, USA.

| Model | AIC | ∆AIC | *w* | Deviance |
| --- | --- | --- | --- | --- |
| Site(intercept) | 223.8 | 0.0 | 0.37 | 219.8 |
| Treatment + Site(intercept) | 224.4 | 0.6 | 0.27 | 218.4 |
| Season + Site(intercept) | 224.4 | 0.6 | 0.27 | 218.4 |
| Treatment * Season + Site(intercept) | 226.8 | 3.0 | 0.08 | 216.8 |

^†^mesopredator exclosures and controls

^‡^warm and cool

Table S3. Four generalized linear mixed-effects models (GLMM) were constructed with an exponential distribution constructed to analyze differences in carcass removal times as a function of treatment^a^ and season^b^ based on carcass trials completed in July 2014 – June 2015 at Ichauway in southwest Georgia, USA.

| Model | AIC | ∆AIC | *w* | Deviance |
| --- | --- | --- | --- | --- |
| Season + Site(intercept) | 163.5 | 0.0 | 0.88 | 155.5 |
| Treatment * Season + Site(intercept) | 167.4 | 3.9 | 0.12 | 155.4 |
| Site(intercept) | 200.5 | 37.0 | 0.00 | 194.5 |
| Treatment + Site(intercept) | 202.5 | 39.0 | 0.00 | 194.5 |

^†^mesopredator exclosures and controls

^‡^warm and cool
